# Pathological stratification and therapeutic implications for post-stroke depression based on a multidimensional biomarker panel: a narrative review

**DOI:** 10.3389/fneur.2026.1810716

**Published:** 2026-06-24

**Authors:** Xinyu Jiang, Yujia Fan, Yizhi Cui, Cui Li, Gang Liu

**Affiliations:** 1 Heilongjiang University of Traditional Chinese Medicine, Harbin, China; 2 Second Affiliated Hospital, Heilongjiang University of Chinese Medicine, Harbin, China; 3 Fourth Affiliated Hospital, Harbin Medical University, Harbin, China

**Keywords:** biomarkers, multi-target modulation, post-stroke depression, provisional phenotypes, therapeutic implications

## Abstract

Post-stroke depression (PSD) severely impedes neurological recovery and remains difficult to manage using a serotonin-centered treatment paradigm alone. This review summarizes evidence suggesting that PSD arises not from a single neurotransmitter deficiency, but from coupled dysregulation across multiple biological dimensions—including monoamines, neuropeptides, neurotrophic factors, and immune-inflammatory mediators—within and beyond the dorsal raphe nucleus–medial prefrontal cortex (DRN–mPFC) circuit. On this basis, we outline a provisional biomarker-informed phenotypic framework for PSD, including the “low-monoamine phenotype,” “high inflammatory burden phenotype”, and “neuropeptide-dominant phenotype.” We further discuss the potential therapeutic implications of this framework and the possible value of multimodal biomarkers for risk stratification and mechanism-guided management. However, these phenotype-treatment links remain preliminary and should be interpreted according to the strength of available evidence, with PSD-specific clinical evidence prioritized over indirect evidence from major depressive disorder and animal/mechanistic studies. This review therefore provides an evidence-informed conceptual framework for future biomarker-guided research in PSD rather than formal treatment recommendations.

## 1 Introduction

PSD is the most common neuropsychiatric complication after stroke, with an overall 5-year prevalence of approximately 30% (1, 2). It not only impedes neurological recovery but also increases medical burden and long-term care needs (3). Current guidelines primarily recommend selective serotonin reuptake inhibitors (SSRIs) as first-line treatment. However, this single-target strategy has important limitations: approximately 30–50% of patients show an inadequate response, the onset of action is often delayed, and some patients discontinue treatment because of poor tolerability (4). These observations suggest that PSD is biologically heterogeneous and that simply increasing synaptic serotonin (5-hydroxytryptamine, 5-HT) is insufficient for all patients. Biomarker-informed stratification may therefore provide a useful framework for improving risk identification and guiding future individualized management. Rather than assuming that all patients respond similarly to serotonin-centered treatment, it may be useful to consider provisional biomarker-informed phenotypes—such as a low-monoamine phenotype, a high inflammatory burden phenotype, and a neuropeptide-dominant phenotype—as research-oriented categories for conceptualizing heterogeneity in PSD (5, 6). These phenotypes are not proposed as validated clinical treatment categories. In addition, because stroke is itself a complex systemic disorder involving inflammatory, neural, and metabolic disturbances, candidate biomarkers in post-stroke depression (PSD) should be interpreted cautiously with regard to specificity and sensitivity (7, 8). At present, these biomarkers are more appropriately viewed as tools for risk stratification and biological characterization rather than as stand-alone diagnostic substitutes for clinical assessment. Diagnosis of depression still relies primarily on structured clinical evaluation, while biomarkers may contribute complementary multidimensional information. Instead, they serve as a framework for discussing how different biological patterns may relate to candidate therapeutic directions. For example, inflammation-predominant features may support interest in anti-inflammatory augmentation, whereas impaired neuroplasticity may justify further study of interventions targeting the brain-derived neurotrophic factor (BDNF)/cAMP response element-binding protein (CREB) pathway or broader network plasticity (9, 10).

## 2 Objective of this review

The objective of this review was to summarize current evidence on multidimensional biomarkers and related mechanisms in post-stroke depression (PSD), with particular emphasis on monoaminergic dysfunction, neuropeptide alterations, neurotrophic signaling, and immune-inflammatory dysregulation. We also examined the potential value of these biomarker dimensions for early risk identification and provisional phenotypic stratification, while considering the impact of key clinical confounders and stroke-related heterogeneity on biomarker interpretation. In addition, we discussed their possible therapeutic implications within an evidence-graded framework. Rather than providing formal treatment recommendations, this article was intended to offer an evidence-informed conceptual synthesis and to outline future directions for biomarker-guided research and mechanism-informed management in PSD.

## 3 Methods

### 3.1 Search strategy and data sources

This article was conducted as a narrative review informed by a structured literature search. The search strategy was developed according to the predefined thematic focus of the manuscript, namely PSD, serotonergic dysfunction, neuropeptides, neurotrophic signaling, inflammatory pathways, biomarker detection, and biomarker-informed phenotypic stratification.

A structured literature search was performed in PubMed/MEDLINE, Web of Science, Embase, and the Cochrane Library for studies published up to December 15, 2025. In addition, the reference lists of included studies and relevant reviews were manually screened to identify further articles of relevance.

Representative search terms included combinations of the following keywords: “post-stroke depression” OR “PSD,” “stroke” AND “depression,” “serotonin” OR “5-HT” OR “monoamine,” “neuropeptide” OR “substance P” OR “SP” OR “CCK-8” OR “neuropeptide Y” OR “NPY” OR “CRH/CRF,” “brain-derived neurotrophic factor” OR “BDNF” OR “CREB,” “inflammation” OR “CRP” OR “IL-6” OR “TNF-*α*,” “biomarker,” “phenotype” OR “subtype” OR “biotype,” and “precision therapy” OR “stratified intervention.” These terms were used alone and in combination, depending on the syntax requirements of each database.

### 3.2 Eligibility criteria

Studies are considered eligible if they meet one or more of the following criteria: (1) involve patients with PSD or stroke survivors at risk of PSD; (2) investigate biomarkers related to monoaminergic signaling, neuropeptides, neurotrophic factors, inflammatory mediators, or related multimodal indicators; (3) examine the association of these biomarkers with PSD occurrence, symptom severity, prognosis, risk identification, or treatment response; or (4) provide mechanistic or translational evidence relevant to the multidimensional biomarker framework discussed in this review.

Original clinical studies, randomized controlled trials, observational studies, systematic reviews, meta-analyses, and highly relevant animal or mechanistic studies are considered for inclusion, because the present review aims to integrate both PSD-specific evidence and biologically informative supporting data. Studies are excluded if they are clearly unrelated to PSD, lack relevance to the principal biomarker axes emphasized in this review, or provide insufficient methodological information for meaningful interpretation.

### 3.3 Date and language restrictions

The search was limited to articles published in English. Studies published up to December 15, 2025 were considered. No formal lower date limit was imposed, because this review included both foundational mechanistic studies and more recent biomarker-oriented clinical studies relevant to PSD.

### 3.4 Study selection

Retrieved records were screened in two stages. First, titles and abstracts were reviewed for relevance to PSD, biomarker dimensions, underlying pathogenic mechanisms, and potential therapeutic implications. Second, full texts of potentially relevant articles were assessed according to the predefined thematic scope and eligibility criteria of the review.

When multiple studies addressed similar biological pathways or therapeutic concepts, priority was given to those most directly related to PSD, particularly PSD-specific clinical studies, followed by highly relevant depression or mechanistic studies used to support biological interpretation where PSD-specific evidence remained limited.

### 3.5 Quality appraisal and risk-of-bias considerations

Given the narrative nature of this review and the heterogeneity of the included literature, a formal pooled risk-of-bias analysis was not performed. Instead, the included evidence was appraised qualitatively according to study design, relevance to PSD, clarity of biomarker assessment, and consistency with other available findings.

Greater interpretive weight was assigned to PSD-specific clinical studies, randomized trials, and meta-analyses. By contrast, animal experiments and indirect evidence from broader depression research were interpreted more cautiously and were used primarily to support biological plausibility and hypothesis generation rather than definitive clinical inference.

### 3.6 Evidence synthesis

Because of substantial heterogeneity in study design, populations, biomarkers, detection methods, and reported outcomes, no quantitative meta-analysis was undertaken. Instead, a narrative synthesis was performed.

The evidence was organized into four principal domains consistent with the structure of the manuscript: (1) monoaminergic dysregulation, particularly serotonergic signaling; (2) neuropeptide alterations; (3) neurotrophic dysfunction, particularly BDNF/CREB-related pathways; and (4) immune-inflammatory activation.

Within each domain, findings were synthesized according to their relevance to PSD and their contribution to the proposed multidimensional biomarker framework. For interpretive clarity, evidence related to phenotypic stratification and treatment implications was considered across four inferential levels: (1) PSD-specific randomized controlled trials or meta-analyses; (2) PSD-specific observational, prognostic, or biomarker association studies; (3) indirect clinical evidence from major depressive disorder or broader depression populations; and (4) animal or mechanistic evidence. Throughout the review, these evidence types were not treated as equivalent. Instead, PSD-specific interventional evidence was given the greatest weight for clinical interpretation, whereas indirect or mechanistic evidence was used primarily to support biological plausibility, provisional extrapolation, and future research hypotheses.

## 4 Pathological mechanisms of PSD

### 4.1 Structural and functional circuits

The dorsal raphe nucleus (DRN) serves as the core origin of the central 5-hydroxytryptamine (5-HT) system. Its tryptophan hydroxylase 2 (TPH2)-positive neurons project to the medial prefrontal cortex (mPFC) via ascending midbrain–forebrain pathways, maintaining the homeostatic balance between excitatory and inhibitory neurons in emotional regulation (11). Under PSD conditions, the functional coupling of this pathway is impaired, resulting in reduced 5-HT levels within the mPFC and concomitant emotional disturbances (12). Multiple animal studies have shown reduced number or activity of DRN serotonergic neurons together with lower 5-HT levels in the mPFC (13). This dual disruption impairs the ascending DRN–mPFC monoaminergic pathway and contributes to an imbalance between excitatory glutamatergic and inhibitory GABAergic activity, thereby promoting emotional and cognitive dysfunction. Consistent with this view, chemogenetic activation of DRN serotonergic neurons restores excitatory-inhibitory balance in the mPFC and ameliorates emotional dysfunction, whereas ablation of the DRN abolishes the antidepressant effects of electroacupuncture (3). In the chronic social defeat stress (CSDS) model, depression-like mice exhibit reduced activity of serotonergic neurons in the DRN and insufficient baseline 5-HT levels in the mPFC, accompanied by social avoidance and decreased cognitive flexibility. Activation of astrocytes in the DRN can restore calcium signaling coupling along the DRN → mPFC pathway, enhance 5-HT transmission, and reverse the aforementioned behavioral deficits (14). Mice with adolescent-specific deletion of the 5-HT₁a heteroreceptor in the mPFC, even without exposure to significant stress in adulthood, display persistent depression-like behaviors and impaired cognitive adaptability, concurrent with high baseline 5-HT levels but blunted stress responses in the mPFC, and low baseline 5-HT levels yet exaggerated stress responses in the DRN. These findings highlight the critical role of adolescent mPFC–DRN 5-HT signaling in lifelong emotional and cognitive homeostasis (15). Additional optogenetic studies have demonstrated that inhibition of DRN 5-HTergic neurons impairs glutamate-dependent long-term potentiation (LTP) in the mPFC and leads to reduced performance in working memory and attention tasks. Conversely, selective activation of this pathway enhances emotional resilience and improves cognitive performance (16, 17). Collectively, these lines of evidence indicate that dysfunction of the DRN–mPFC serotonergic pathway constitutes a core basis for the emotional and cognitive impairments observed in PSD. However, what underlies the decline in circuit function? Elucidating the underlying synaptic and molecular mechanisms will facilitate a fundamental understanding and precise correction of this abnormality.

### 4.2 Reduction in 5-HT levels

While establishing that the DRN → mPFC circuit is impaired, elucidating the causes underlying the decline in circuit function requires a deeper investigation into the synaptic and molecular mechanisms. Further dissection of “true deficiency” and “functional exhaustion” at the synaptic level will facilitate the selection of more targeted intervention strategies. Synaptic 5-HT depletion encompasses two distinct forms: one is quantitative insufficiency, primarily caused by reduced synthesis or release; the other is diminished efficacy, characterized by impaired signal transmission despite relatively unchanged 5-HT concentrations. These two forms often coexist and interact. First, the activity of the serotonin transporter (SERT) serves as a key regulatory node. Excessively high SERT activity accelerates 5-HT reuptake, leading to persistently low levels of available 5-HT in the synaptic cleft (18, 19). However, when the rate of reuptake exceeds that of release, depletion dominated by functional insufficiency emerges. Clinically, the use of SSRIs to increase synaptic 5-HT availability by inhibiting reuptake provides indirect evidence for this mechanism. Second, structural and functional alterations in receptor subtypes can also lead to reduced efficacy of synaptic signaling. Even if 5-HT concentrations are not significantly decreased, diminished expression, sensitivity, or signal coupling efficiency of key receptors such as 5-HT₁a, 5-HT₂A, and 5-HT₂C can attenuate the response of neural networks to the same concentration of 5-HT, thereby resulting in depressed mood and reduced cognitive flexibility (16, 20, 21).

Third, lesion-related damage can trigger “true deficiency.” As the central hub for 5-HT synthesis and projection, the DRN exhibits a marked reduction in central 5-HT synthesis and release once TPH2 is inhibited or 5-HT neurons are lost. Moreover, structural or functional impairment in projection target regions such as the prefrontal cortex can also disrupt normal signal reception (12, 22, 23). This phenomenon is more commonly observed in post-stroke depression and several neurodegenerative disorders (12, 24).

### 4.3 Integrated regulation of the 5-HT circuit by neuropeptides, neurotrophic factors, and inflammatory factors

Emotional and cognitive regulation in PSD extends beyond the 5-HT pathway alone and involves extensive crosstalk across multiple molecular systems. Neuropeptides such as CCK-8 and substance P can modulate serotonergic signaling by altering synaptic release and receptor activation dynamics (25, 26). In parallel, BDNF and CREB regulate serotonergic neuron function, synaptic plasticity, and related gene expression (27–29). Inflammatory mediators such as IL-6 and TNF-*α* may further impair serotonergic signaling by altering tryptophan metabolism and downregulating receptors or transporters (30–32). Together, these interactions support the biological rationale for multi-target approaches in PSD, although current support remains heterogeneous and should be interpreted cautiously. From a contemporary perspective, monoaminergic dysfunction should not be viewed as a sufficient stand-alone explanation for PSD, but rather as one component of a broader pathological network involving neuropeptide signaling, neuroplasticity, immune-inflammatory activation, and circuit-level dysregulation.

## 5 Detection of 5-HT and biomarkers

Detection of 5-HT in the periphery and central nervous system (CNS) actually represents two distinct physiological systems. In the periphery, 5-HT is primarily derived from enterochromaffin cells in the gut (33), synthesized with the involvement of tryptophan hydroxylase 1 (TPH1), and coordinately regulated by processes including storage, release, transport, and metabolism mediated by monoamine oxidase (MAO) and other enzymes. It is therefore more susceptible to alterations in endocrine or immune status and often fails to directly reflect synaptic levels within the brain. In contrast, central 5-HT detection typically relies on invasive cerebrospinal fluid (CSF) sampling or indirect estimation via surrogate markers such as receptors and transporters. Such approaches not only struggle to capture instantaneous synaptic concentrations but also lack precise spatial resolution for specific circuits. These differences in source and pathway introduce inherent biases in the correspondence between peripheral and central 5-HT levels (34, 35). At the analytical level, sensitivity and specificity represent two major challenges. Regarding sensitivity, 5-HT exhibits rapid turnover and is readily degraded by MAO-A (36, 37). Platelet activation during sampling can trigger instantaneous 5-HT release, leading to artificially elevated readings (38). Furthermore, low concentrations and a short half-life increase the risk of missed detection, mandating strict control of pre-analytical variables and maximal improvement of detection sensitivity. With respect to specificity, absolute concentration does not equate to “effective signaling.” Variations in the expression and sensitivity of receptor subtypes, as well as the state of circuit plasticity, can lead to divergent physiological outcomes from the same 5-HT concentration. Meanwhile, crosstalk between 5-HT and immune, metabolic, and other monoaminergic systems further complicates interpretive context, rendering a single concentration metric inadequate as a definitive disease biomarker (39, 40). Accordingly, peripheral 5-HT should be interpreted as a surrogate biochemical snapshot rather than a direct readout of central serotonergic tone. For studies intending to use peripheral 5-HT in biomarker-informed stratification, sampling conditions should be standardized as much as possible, including consistent timing of blood collection, minimization of platelet activation during handling, prompt processing, and controlled storage conditions to reduce degradation-related variability. Even under standardized conditions, peripheral 5-HT should not be used in isolation to assign a phenotype, but rather interpreted together with inflammatory, neurotrophic, neuropeptide, and clinical features.

## 6 Multi-target mechanistic intervention strategies based on 5-HT

### 6.1 Interventional effects of SSRIs

SSRIs are the most commonly used first-line medications in the current treatment of PSD (12, 41, 42). They act by inhibiting the presynaptic reuptake of 5-HT, thereby prolonging the duration of neurotransmitter action in the synaptic cleft and alleviating emotional symptoms. Multiple randomized controlled trials (RCTs) and systematic reviews have demonstrated that commonly used SSRIs, including fluoxetine, sertraline, citalopram, and paroxetine, significantly reduce scores on the Hamilton Depression Rating Scale (HAMD) during both the acute and recovery phases, with some studies also reporting concomitant improvements in cognitive function and activities of daily living. In a multicenter RCT conducted by Robinson et al. (43), escitalopram was further shown not only to ameliorate depressive symptoms in stroke patients but also to significantly reduce the incidence of PSD, with favorable overall tolerability. A recent Cochrane systematic review (41), after synthesizing data from multiple RCTs, confirmed that SSRIs are associated with higher remission rates of depressive symptoms in stroke patients compared with placebo, and systematically summarized their common adverse effect profiles. A network meta-analysis (44) that further compared the relative effects of different antidepressants on HAMD improvement indicated that SSRIs rank highly in terms of short-term efficacy, particularly in patients during the acute stroke phase. The overall short-term efficacy of SSRIs is relatively favorable, with particularly prominent performance in patients with acute stroke. However, the therapeutic efficacy of SSRIs may be blunted in patients with concurrent persistent inflammation, insufficient neurotrophic factor levels, or disrupted neuropeptide signaling (45, 46).

### 6.2 Limitations of SSRI therapy

The most common adverse reactions to SSRIs include gastrointestinal discomfort, insomnia, decreased sexual function, emotional blunting, and an elevated bleeding risk associated with the inhibition of platelet function (47). Systematic reviews suggest that the use of SSRIs may increase the risk of stroke-related hemorrhagic events, requiring particular caution, especially in patients receiving concurrent antiplatelet or anticoagulant therapy (48). Furthermore, elderly stroke patients exhibit relatively poor tolerability to SSRIs. Agents such as paroxetine are more susceptible to altered pharmacokinetics in this population, thereby increasing the risk of adverse reactions and drug–drug interactions (49). Collectively, SSRIs have well-established short-term efficacy in the treatment of PSD, but their tolerability and safety require careful individualized assessment, particularly in older patients and in those receiving concurrent antiplatelet or anticoagulant therapy. In patients with elevated inflammatory burden or impaired neuroplasticity, interventions targeting only the 5-HT pathway may be insufficient to fully address emotional and cognitive disturbances. These considerations support further exploration of biomarker-informed and mechanism-guided management approaches in PSD, although the clinical utility of such stratification remains to be prospectively validated.

## 7 Future directions: precision intervention and stratified therapy

### 7.1 Comparison of early PSD biomarkers: monoamines, neuropeptides, neurotrophic factors, and inflammatory factors

In studies of early-stage PSD, CCK-8, substance P (SP), and 5-HT are among the most frequently evaluated molecular markers (26). Clinical and animal data suggest that these markers differ in both biological fluctuation and detectability during the early stage of disease (50, 51). SP levels tend to rise with increasing PSD severity, whereas peripheral 5-HT shows marked acute-phase fluctuation and a weaker correlation with depression scale scores (2, 52). CCK-8 may change before overt symptom onset and appears to show a stronger relationship with disease progression than 5-HT (25). Mechanistic studies further suggest that neuropeptide changes may precede monoamine depletion under conditions of stress-related inflammation or gut dysbiosis (53). From an analytical perspective, peripheral 5-HT is less stable and more vulnerable to timing and transport-related interference (36), whereas CCK-8 and SP may show relatively more stable enzyme-linked immunosorbent assay (ELISA)-based signals. Positron emission tomography (PET) receptor imaging provides complementary information on central receptor status, although its clinical use in acute stroke remains limited by cost and accessibility (54, 55). Overall, current evidence suggests that CCK-8 and SP may be more sensitive than peripheral 5-HT for early PSD-related biological detection (50–52).

In the early screening of PSD, single biomarkers are prone to missed detection. The integration of the serotonergic, neuropeptide, neurotrophic, and inflammatory axes into a “multi-axis panel” tends to yield higher sensitivity and robustness. Studies in acute stroke have demonstrated that serum BDNF is significantly associated with the occurrence of PSD and can be used for risk prediction; combining low BDNF levels with clinical features further improves predictive performance (45). In a cohort of 70 stroke patients, CCK-8, SP, and 5-HT were all correlated with depression severity, with the strength of Spearman correlations ranked as CCK-8 > SP > 5-HT, suggesting that neuropeptide markers may be more sensitive than 5-HT for the early identification of PSD (50). Beyond the differences between neuropeptides and monoamines, neurotrophic factors also demonstrate significant value in the early detection of PSD. As a key regulator of synaptic plasticity and neurogenesis, pooled analyses of multiple studies have shown that peripheral BDNF levels are significantly lower in patients who develop subsequent PSD during the early stroke phase, indicating that BDNF may be used to identify individuals at high risk of future PSD (46). In contrast to single monoamine markers, which rely solely on peripheral neurotransmitters and are thus vulnerable to various peripheral confounding factors, BDNF—an indicator of neurotrophic status—exhibits consistent associations with subsequent PSD onset across multiple studies. Therefore, combining BDNF with monoamine and neuropeptide markers is expected to enhance the robustness of early risk identification. Evidence from the inflammatory axis indicates that post-stroke PSD is associated with peripheral immune activation. This association should be interpreted within the broader context that stroke itself is a systemic inflammatory event. Accordingly, inflammatory biomarkers may capture both stroke-related tissue injury and depression-related biological vulnerability, which is why they are better suited to risk stratification than to stand-alone diagnostic use. A meta-analysis of C-reactive protein (CRP) levels in the acute phase revealed higher CRP concentrations on admission in patients who developed PSD, with significant differences persisting in the subgroup evaluated for depression at ≥1 month after stroke (56). Another review also reported higher CRP levels in the PSD group, which were correlated with the risk of depression onset (57). Cytokine evidence further demonstrates elevated pro-inflammatory factors such as IL-6 in PSD patients, with IL-6 levels associated with PSD at multiple time points, including the acute phase, discharge, and follow-up (58–60). Collectively, these findings indicate that incorporating serotonin, neuropeptides, BDNF, and inflammatory factors into a unified panel creates complementary signals. Such a panel covers both inflammatory and neurotrophic foundations while reflecting early changes in chemical signaling and emotional regulation, rendering it more reliable for early PSD prediction than single biomarkers alone. From a practical perspective, a provisional biomarker framework in PSD may be conceptualized at two levels. A clinically more accessible core set may include inflammatory markers such as CRP and IL-6, neurotrophic indicators such as BDNF, and cautiously interpreted peripheral 5-HT under standardized pre-analytical conditions. By contrast, neuropeptide measures, exosome-related markers, and advanced receptor imaging are better regarded as extended or research-enriching indicators that may refine biological interpretation but are not yet sufficiently standardized for routine phenotype assignment.

### 7.2 Confounding factors and sources of heterogeneity in biomarker-informed PSD stratification

Biomarker-informed stratification in PSD should be interpreted in the context of substantial biological and clinical heterogeneity (1, 2, 12, 42). Candidate biomarker profiles may be influenced not only by depressive liability, but also by stroke-related factors such as stroke subtype, lesion location, stroke severity, and time since stroke; patient-related factors including age, frailty, multimorbidity, and pre-stroke psychiatric vulnerability; treatment-related factors such as exposure to SSRIs or SNRIs, antiplatelet or anticoagulant therapy, anti-inflammatory treatment, and rehabilitation intensity; as well as measurement-related factors, particularly pre-analytical and assay-related variability for peripheral 5-HT (36). In addition, symptom overlap between depression and other post-stroke manifestations, including fatigue, apathy, sleep disturbance, and cognitive slowing, may further complicate phenotype interpretation (42, 61). Therefore, provisional phenotype assignment in PSD should be regarded as a context-dependent, multidimensional research construct rather than a direct substitute for formal clinical diagnosis, and future studies should explicitly model these confounders when evaluating reproducibility, predictive validity, and treatment relevance.

### 7.3 Multi-target modulation: 5-HT, neuropeptides, neurotrophic factors, and inflammation

Conventional single-target therapies such as SSRIs primarily act by inhibiting 5-HT reuptake and increasing synaptic serotonin levels. However, increasing 5-HT alone may be insufficient to restore the emotional and cognitive functions impaired after stroke. Animal experiments and selected clinical studies therefore suggest that multi-target modulation may also be relevant in PSD. Such approaches may involve not only enhancement of 5-HT availability but also modulation of BDNF and downstream CREB signaling to support synaptic plasticity and network recovery (58). Any therapeutic implication discussed in this section should be interpreted in light of the confounding factors outlined above and the inferential hierarchy defined in the Methods. Activation of the BDNF/CREB pathway is particularly critical in brain regions including the hippocampus and prefrontal cortex. It is closely associated with the reconstruction of synaptic structures and the survival of newborn neurons, facilitates the induction of LTP, and thereby improves the recovery of emotional regulation and cognitive function (62). For instance, Wu et al. (63) found in a mouse model of PSD that Chaihu Shugan San could regulate inflammatory responses via an exosome-mediated miRNA pathway, simultaneously elevate 5-HT and BDNF levels, upregulate the expression of CREB and its phosphorylated form p-CREB, and ultimately significantly ameliorate depression-like behaviors and exploratory activity. Furthermore, Jin et al. (64)demonstrated that fluoxetine in PSD models not only increases brain 5-HT concentrations but also promotes the phosphorylation of the transcriptional regulator methyl-CpG-binding protein 2 (MeCP2) through a PKA-dependent mechanism, releasing its inhibition of the BDNF IV promoter and thus upregulating the CREB-BDNF signaling pathway. This finding suggests that even conventional SSRIs may exert synergistic effects through multiple molecular pathways, thereby achieving more comprehensive and durable therapeutic outcomes.

PSD is a complex syndrome arising from the combined effects of neuroinflammation, monoamine neurotransmitter imbalance, and diminished synaptic plasticity. The acute inflammatory response following stroke not only disrupts the blood–brain barrier but also activates microglia and astrocytes, inducing the sustained release of pro-inflammatory factors including IL-1β, IL-6, and TNF-*α*, thereby establishing a chronic neuroinflammatory milieu. Persistently active inflammatory signaling can suppress BDNF–CREB transduction in the hippocampus and prefrontal cortex via pathways such as nuclear factor kappa B (NF-κB) activation, thereby weakening synaptic plasticity—a mechanism recognized as a key contributor to the development of depressed mood and cognitive impairment (65). Conventional antidepressants such as SSRIs act primarily by modulating the 5-HT system but often exhibit limited efficacy in patients with inflammation-driven PSD (66). At the evidence level, the association between inflammatory activation and PSD risk is supported mainly by PSD-specific observational and meta-analytic biomarker studies, whereas most interventional evidence for anti-inflammatory augmentation derives from major depressive disorder or mixed depression populations rather than PSD-specific randomized trials (56, 59, 67). For example, the traditional Chinese medicine Chaihu Shugan San can suppress inflammation by downregulating the Toll-like receptor 4 (TLR4)/NF-κB pathway while simultaneously upregulating 5-HT and BDNF levels, resulting in significant improvements in depression-like behaviors in animal models of PSD (63). Recent experimental findings and limited clinical evidence suggest that anti-inflammatory augmentation may help attenuate relevant pathological processes in selected patients by reducing inflammatory signaling, potentially relieving inhibition of the BDNF–CREB pathway and supporting neuronal plasticity (63, 67). Furthermore, minocycline can alleviate emotional and cognitive dysfunction in ischemic stroke models by inhibiting microglial activation, reducing the release of IL-1β and TNF-*α*, and promoting the expression of p-CREB and BDNF (68). Supportive clinical evidence has also emerged for anti-inflammatory approaches in stroke and depression populations, although direct PSD-specific clinical validation for minocycline remains limited (67, 69–71). Recent systematic reviews and randomized controlled trials demonstrate that anti-inflammatory agents, as adjunctive therapy to SSRIs, not only accelerate the improvement of mood in post-stroke patients but also enhance quality of life (67). Accordingly, anti-inflammatory intervention in PSD should currently be interpreted as a PSD-modified biological hypothesis informed by mechanistic and indirect clinical evidence, rather than as a phenotype-validated treatment standard.

Neuropeptides play pivotal roles in emotional regulation, stress responses, and the maintenance of synaptic plasticity, among which neuropeptide Y (NPY), substance P (SP), and CRF are the most extensively studied (72). Studies have shown that peripheral blood levels of NPY are abnormal in patients with PSD, with levels closely correlated with depression severity and prognosis; NPY gene expression may serve as an independent predictor of PSD (73). Within the central nervous system, NPY is most abundant in the hippocampus, followed by the cerebral cortex, hypothalamus, thalamus, brainstem, and cerebellum. This distribution pattern suggests that NPY mediates specific physiological functions in emotional regulation and post-stroke recovery. NPY is widely implicated in immune modulation, inflammatory responses, and the preservation of synaptic plasticity. During the course of stroke, dynamic changes in serum NPY levels are recognized as representative biomarkers, closely associated with the occurrence of stroke-related complications. Its associated signaling pathways demonstrate potential clinical value for diagnosis, prognostic evaluation, and therapeutic intervention (74). At the evidence level, PSD-specific support for neuropeptide involvement is currently strongest for biomarker association and mechanistic plausibility, whereas direct interventional validation in PSD remains sparse. Several therapeutic implications cited in this field are extrapolated from animal studies, translational models, or major depressive disorder rather than PSD-specific randomized trials (74–77). Regarding interventional studies, the traditional Chinese medicine formula Xiaoyao Jieyu San has shown favorable efficacy in alleviating PSD symptoms in both animal and clinical trials. Its mechanism of action may involve regulation of the hypothalamic–pituitary–adrenal (HPA) axis, reduction of pro-inflammatory factor levels, and upregulation of neuropeptide expression, highlighting the advantages of comprehensive multi-target intervention (77). Furthermore, corticotropin-releasing factor (CRF), a neuropeptide closely linked to stress, has also been implicated in the pathogenesis of PSD. Studies have revealed that in PSD models, CRF modulates hippocampal synaptic plasticity by regulating inflammatory responses, oxidative stress, and autophagic activity. Abnormal CRF levels and disrupted receptor signaling represent key factors triggering depression-like behaviors, and blockade of this pathway is considered capable of reversing the pathological state of PSD (78). Taken together, multi-target modulation strategies may offer a useful conceptual direction for PSD by addressing inflammation, neuropeptide imbalance, and impaired synaptic plasticity simultaneously. At present, however, these strategies should be regarded as hypothesis-generating rather than as validated standards of care.

### 7.4 Evidence-graded discussion of a provisional biomarker-informed phenotypic framework in PSD

Based on the evidence summarized in the preceding sections, this section discusses a provisional biomarker-informed phenotypic framework for PSD rather than proposing a validated biological classification system or a basis for formal treatment recommendations. Current evidence suggests that PSD involves coupled dysregulation across monoaminergic, neuropeptide, neurotrophic, and immune-inflammatory systems, and this biological heterogeneity may partly contribute to variability in treatment response across patients (12, 45, 59, 65). In this context, inflammatory markers (e.g., CRP and IL-6), neurotrophic indicators such as BDNF, cautiously interpreted peripheral 5-HT, and selected neuropeptide-related measures may help inform a provisional and research-oriented framework for biological interpretation and risk stratification in PSD (46, 50, 56, 59, 74). However, these constructs remain hypothesis-generating, because their operational definitions, temporal stability, and predictive value for treatment selection have not yet been prospectively validated in PSD-specific cohorts. Accordingly, any tentative phenotype assignment should be interpreted on the basis of converging clinical and biomarker evidence under standardized sampling conditions, rather than any single laboratory value alone. A more accessible evidential basis may include CRP, IL-6, BDNF, and cautiously interpreted peripheral 5-HT, whereas neuropeptide assays, exosome-related markers, and advanced receptor imaging are better regarded as extended indicators for biological refinement rather than routine clinical allocation (36, 45, 46, 50, 55, 56, 59, 74).

**Figure 1:**
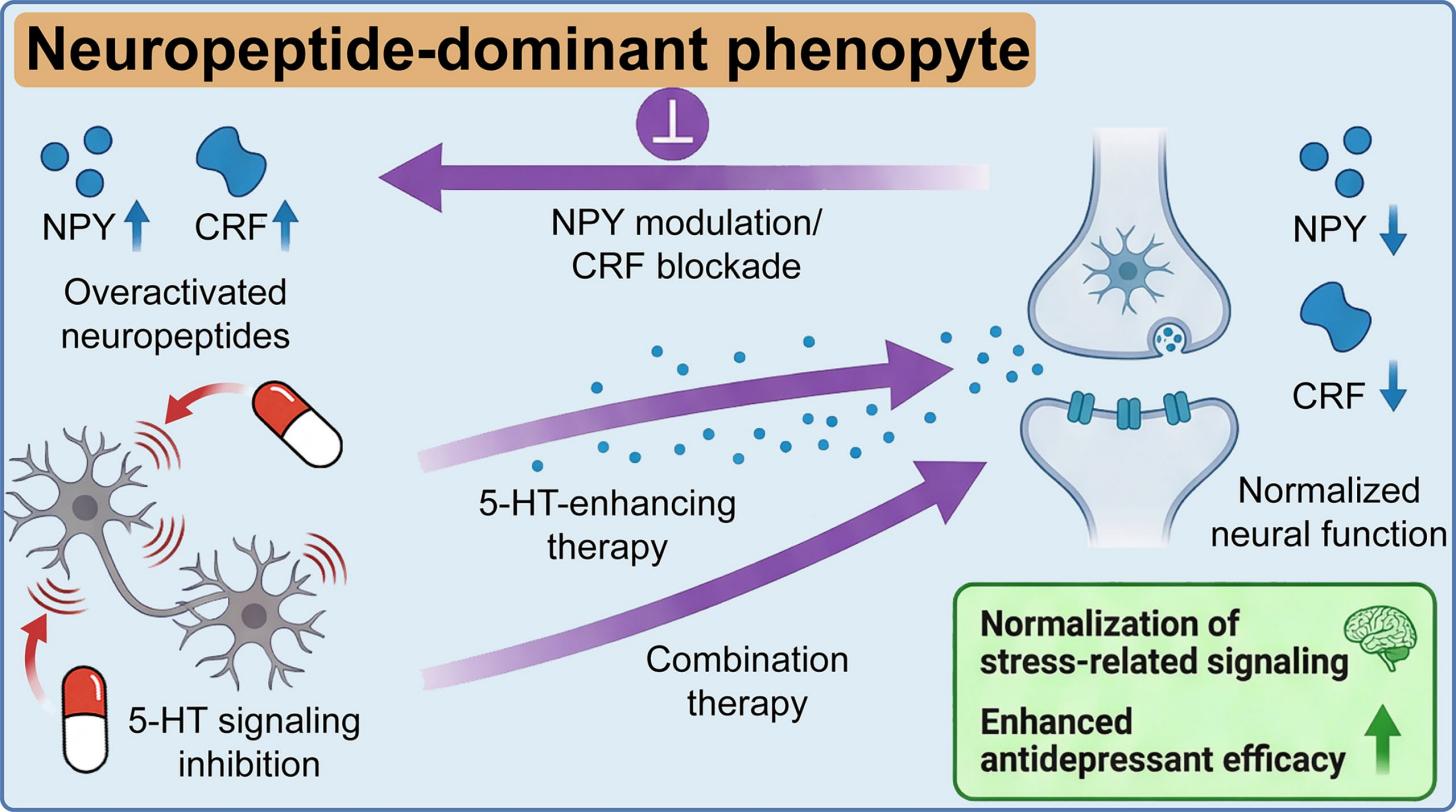
Conceptual schematic of the low 5-HT phenotype in post-stroke depression and its conditional mechanism-informed therapeutic implications. Reduced 5-HT availability and impaired serotonergic signaling are illustrated as putative features of this provisional phenotype. 5-HT, 5-hydroxytryptamine (serotonin).

First, a low-monoamine phenotype may be provisionally considered when relatively reduced serotonergic signal is observed under standardized sampling conditions and when this pattern is not better explained by marked inflammatory activation, lesion-related effects, medication exposure, or major pre-analytical interference (36, 42). Mechanistically, chronic stress and inflammation-related pathways may reduce tryptophan availability and blunt serotonergic function (31, 32). Poor or delayed early response to monoaminergic treatment may be recorded as a supportive retrospective clue, but should not be used as a defining criterion for phenotype assignment. At the evidence level, direct PSD-specific support in this context mainly concerns the overall efficacy of SSRIs in PSD rather than validated treatment stratification for a biomarker-defined low-monoamine phenotype (12, 41, 44). Accordingly, serotonergic treatment may remain a reasonable first consideration in patients with putative low-monoamine features, while rehabilitation and psychological interventions may also be incorporated according to clinical context. In cases with concomitant mild-to-moderate inflammatory activation, adjunctive anti-inflammatory treatment could be considered as a provisional and individualized option, although PSD-specific evidence for this phenotype-linked approach remains limited (67, 70, 79). The clinical interpretation of this phenotype should therefore be regarded as a PSD-oriented working hypothesis supported by mechanistic reasoning and indirect biochemical inference, rather than by a validated PSD-specific cutoff or treatment-response algorithm (Figure 1).

**Figure 2:**
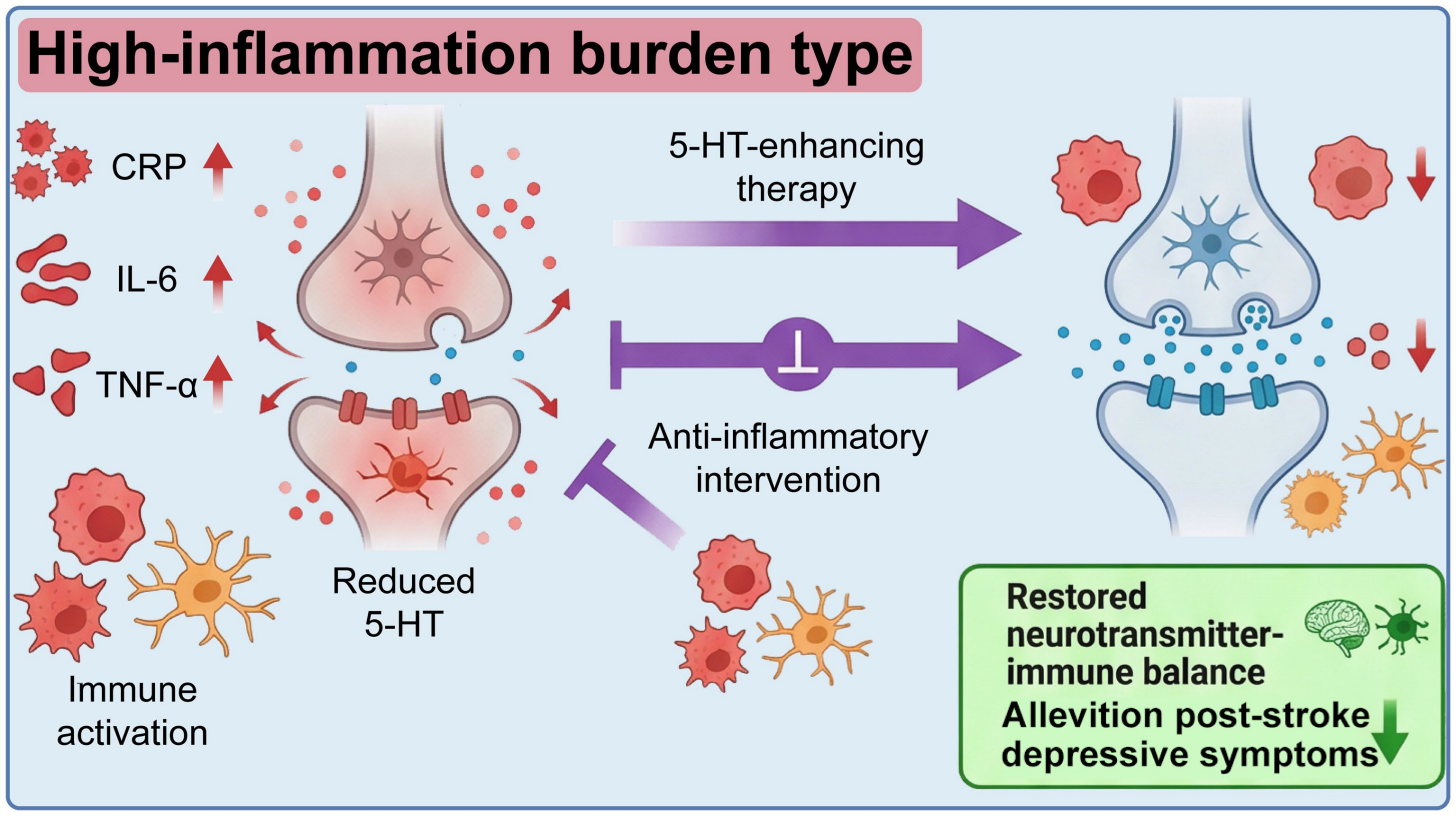
Conceptual schematic of the high-inflammatory-burden phenotype in post-stroke depression and its conditional mechanism-informed therapeutic implications. Elevated inflammatory burden and reduced serotonergic availability are illustrated as putative features of this provisional phenotype. Note: CRP, C-reactive protein; IL-6, interleukin-6; TNF-*α*, tumor necrosis factor-α; 5-HT, 5-hydroxytryptamine (serotonin); PSD, post-stroke depression.

A high inflammatory burden phenotype may be suggested by elevated inflammatory markers such as CRP, IL-6, or TNF-*α*, particularly when accompanied by fatigue, reduced appetite, or decreased physical vitality (5, 6). For interpretive purposes, persistent or repeatedly observed inflammatory elevation beyond the immediate acute injury window may be more informative for PSD-related stratification than a single early post-stroke value alone, because inflammatory markers in the hyperacute phase may reflect tissue injury as much as depressive liability. This interpretation should also take into account concurrent infection, systemic inflammatory disease, concomitant anti-inflammatory medication, and general stroke severity, all of which may amplify or attenuate the apparent inflammatory signal. Current evidence indicates that inflammatory activation is associated with PSD risk, and anti-inflammatory augmentation has shown signals of benefit in broader depression research, especially in subgroups with higher baseline inflammatory burden (67, 79). However, much of this interventional evidence derives from major depressive disorder or mixed depression populations rather than PSD-specific randomized trials. Accordingly, anti-inflammatory approaches in this phenotype should not be presented as established treatment recommendations for PSD, but rather as biologically plausible and evidence-graded options that warrant further prospective evaluation in PSD populations. Thus, the therapeutic implication in this phenotype is best framed as a conditional proposition: in patients with persistent inflammatory elevation and compatible clinical features, anti-inflammatory augmentation may be considered a research-informed option, but only with explicit recognition that the supporting intervention evidence is largely indirect and not yet phenotype-validated in PSD. Combination strategies targeting both inflammation and serotonergic dysfunction may be conceptually attractive, but they should be interpreted as conditional therapeutic hypotheses rather than validated subtype-specific standards of care (Figure 2).

**Figure 3:**
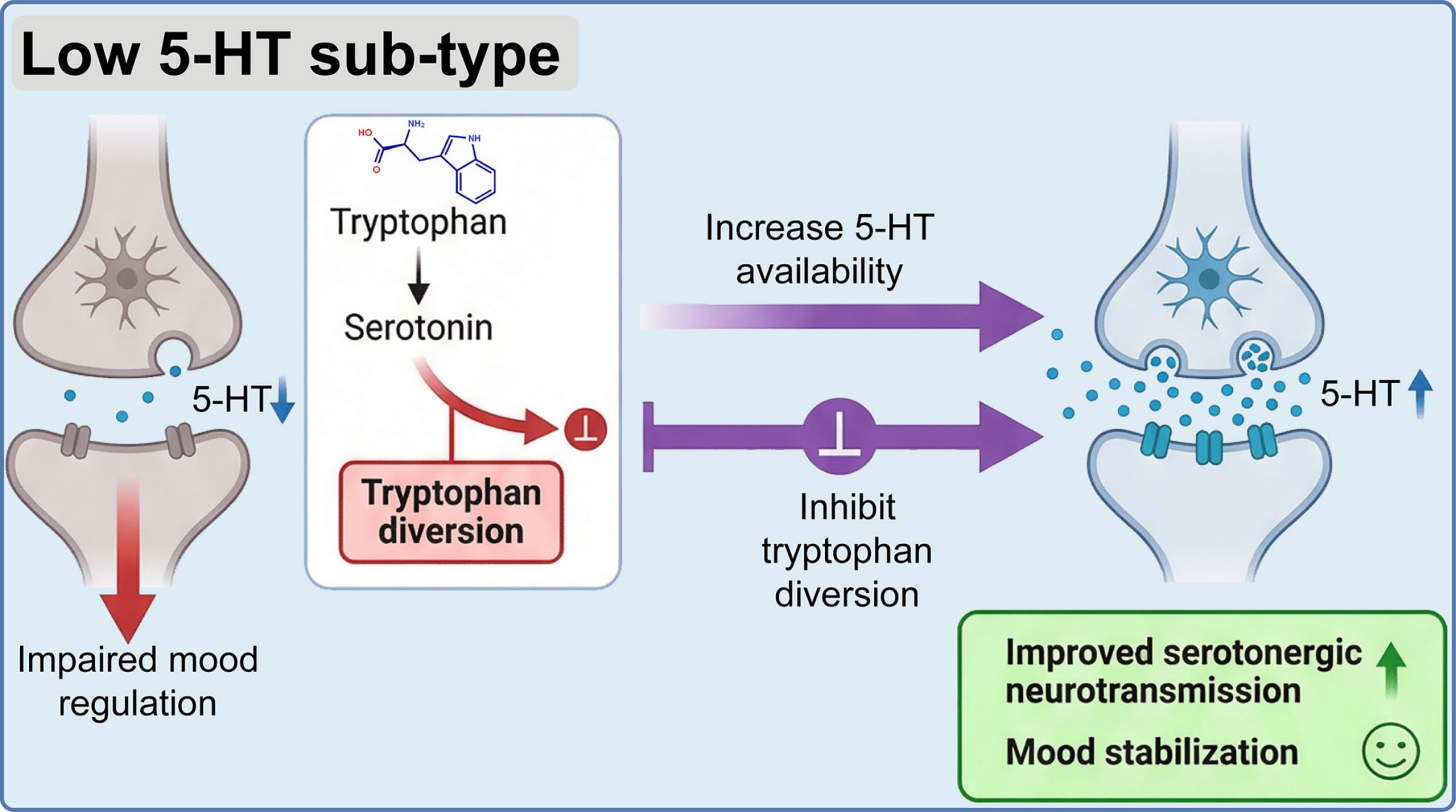
Conceptual schematic of the neuropeptide-dominant phenotype in post-stroke depression and its conditional mechanism-informed therapeutic implications. Dysregulated neuropeptide signaling and impaired serotonergic activity are illustrated as putative features of this provisional phenotype. NPY, neuropeptide Y; CRF, corticotropin-releasing factor; 5-HT, 5-hydroxytryptamine (serotonin); PSD, post-stroke depression.

A neuropeptide-dominant phenotype may involve abnormalities in neuropeptide systems related to stress regulation and emotional processing, including reduced NPY activity and increased CRF signaling. This phenotype may be associated with stress sensitivity, emotional lability, and impaired synaptic plasticity. At present, neuropeptide markers are better interpreted as extended or supportive indicators rather than stand-alone classification tools, because assay standardization, temporal reproducibility, and PSD-specific validation remain insufficient. Existing evidence suggests that neuropeptide pathways are biologically relevant to affective dysregulation and may represent potential therapeutic targets (80). Nevertheless, direct interventional evidence in PSD remains sparse, and some of the currently cited support comes from major depressive disorder or animal experiments rather than PSD-specific clinical trials (75, 76). Therefore, neuropeptide-targeted interventions should be framed cautiously as emerging and largely translational strategies. In evidential terms, this phenotype currently rests more on mechanistic plausibility and associative support than on direct PSD-specific therapeutic validation. Any treatment implication should therefore be regarded as exploratory and conditional, rather than as a basis for treatment allocation in routine PSD care. At present, such approaches may help generate future research directions, but they cannot yet be considered evidence-based phenotype-specific treatments for PSD (Figure 3).

Overall, the proposed phenotype-treatment mapping in PSD should be regarded as provisional and hypothesis-generating rather than as a validated clinical treatment algorithm, and a comparative summary of the candidate indicators, major confounders, supporting evidence, and conditional therapeutic implications is provided in Table 1. In addition, these biomarker profiles and the corresponding provisional phenotypes are unlikely to be static across the course of stroke recovery. Their expression may vary across acute, subacute, and chronic stages, and future studies should examine the temporal stability and stage-specific utility of biomarker-informed phenotyping in PSD. Such studies should also account explicitly for major confounders and effect modifiers, including lesion characteristics, systemic inflammatory conditions, medication exposure, age-related variability, and pre-analytical measurement noise. More specifically, future validation should address at least four questions: whether phenotype assignment is reproducible across repeated measurements; whether these provisional phenotypes predict PSD onset, symptom trajectory, or relapse risk; whether they are associated with differential treatment response; and whether they provide incremental value beyond single biomarkers or conventional clinical scales. Prospective PSD cohorts and stratified trial designs based on baseline biomarker burden may be particularly valuable for testing these questions.

**Table 1:** Comparative summary of provisional biomarker-informed PSD phenotypes, candidate indicators, confounders, and conditional therapeutic implications.

Provisional phenotype	Candidate indicators	Major confounders/effect modifiers	Nature of supporting evidence	Conditional therapeutic implications
Low-monoamine phenotype	Relatively reduced serotonergic signal under standardized sampling conditions; supportive neurotrophic context such as low BDNF when available	Pre-analytical variability in peripheral 5-HT measurement, platelet activation, lesion-related effects, medication exposure, inflammatory activation, and stroke stage	PSD-specific evidence mainly supports the overall efficacy of SSRIs in PSD; indirect support comes from mechanistic and biochemical inference for reduced serotonergic tone; no validated PSD-specific cutoff or treatment-response algorithm is currently available	Serotonergic treatment may warrant consideration within the broader clinical context of PSD management, but no phenotype-specific treatment algorithm has been validated for a putative low-monoamine phenotype. Rehabilitation and psychological interventions remain important components of care according to overall clinical context. When inflammatory activation is also present, anti-inflammatory augmentation may be discussed only as a research-informed and individualized hypothesis, because the supporting interventional evidence remains limited and is derived partly from indirect or non-PSD-specific sources
High inflammatory burden phenotype	Persistent or repeatedly elevated inflammatory markers, such as CRP, IL-6, or TNF-α, particularly when accompanied by fatigue, reduced appetite, or reduced physical vitality	Acute stroke-related tissue injury, systemic infection, inflammatory comorbidities, anti-inflammatory medication, stroke severity, and time since stroke	PSD-specific support is mainly derived from biomarker association and prognostic studies; most intervention-related support for anti-inflammatory augmentation comes from MDD or mixed depression populations; phenotype-stratified PSD treatment trials are lacking	Anti-inflammatory augmentation may be considered a research-informed option in patients with persistent inflammatory elevation and compatible clinical features, but should not be interpreted as an established PSD treatment recommendation. Combination approaches remain conditional therapeutic hypotheses
Neuropeptide-dominant phenotype	Altered neuropeptide-related indicators, such as reduced NPY activity or increased CRF signaling; stress sensitivity, emotional lability, and impaired synaptic plasticity	Assay standardization limitations, temporal variability, broader stress-related biological variation, and overlap with non-specific post-stroke emotional and behavioral changes	PSD-specific support is currently strongest for biomarker association and mechanistic plausibility; additional support comes from translational, animal, and non-PSD depression studies; direct interventional validation in PSD remains sparse	Neuropeptide-targeted strategies should be regarded as exploratory and translational. They may inform future research directions but should not be used as a basis for routine phenotype-specific treatment allocation in PSD

5-HT, 5-hydroxytryptamine; BDNF, brain-derived neurotrophic factor; CRP, C-reactive protein; IL-6, interleukin-6; TNF-α, tumor necrosis factor-α; NPY, neuropeptide Y; CRF, corticotropin-releasing factor; MDD, major depressive disorder; PSD, post-stroke depression; SSRIs, selective serotonin reuptake inhibitors.

The phenotypes listed above are presented as provisional, research-oriented constructs rather than validated clinical categories. The therapeutic implications are conditional and evidence-graded, not formal treatment recommendations.

## 8 Conclusions and future perspectives

The 5-HT pathway occupies a central position in PSD, but it should be interpreted within a broader framework of neuropeptide, neurotrophic, and immune-inflammatory dysregulation (81). A multidimensional biomarker panel, particularly when integrated with clinical and imaging information, may provide a useful basis for future risk stratification and phenotype-oriented research in PSD. Nevertheless, the proposed biomarker-informed phenotypic framework and its therapeutic implications remain provisional and require validation in future PSD-specific prospective studies.
